# Le prolapsus génital néonatal: entité exceptionnelle (à propos d’un cas)

**DOI:** 10.11604/pamj.2016.25.153.9853

**Published:** 2016-11-14

**Authors:** Hind Zhiri, Btissam Fatih, Karam Harou, Abderrahim Aboulfalah, Hamid Asmouki, Abderraouf Soummani

**Affiliations:** 1 Université Cadi Ayyad, Service de Gynécologie Obstétrique, CHU Mohammed VI, Marrakech, Maroc

**Keywords:** Prolapsus génital, néonatal, exceptionnel, Genital prolapse, neonatal, exceptional

## Abstract

Le prolapsus génital est une entité exceptionnelle chez le nouveau-né. Il est le plus souvent associé à des anomalies congénitales du système nerveux central. Nous rapportons le cas d’un nouveau-né à terme ayant un prolapsus génital sans anomalies de système nerveux central a travers lequel nous rapportons la particularité diagnostic et thérapeutique de cette pathologie rarissime.

## Abstract (en)

Neonatal genital prolapse is an uncommon entity. It is usually associated with birth defects of the central nervous system. We here report the case of a full term newborn with a genital prolapse not associated with central nervous system abnormalities in order to highlight the diagnostic and therapeutic particularity of this very rare disease.

## Introduction

Le prolapsus génital est une entité exceptionnelle chez le nouveau-né. Il est le plus souvent associé à des anomalies du système nerveux central dans 82% à 86%, particulièrement les anomalies de fermeture du tube neurale [1]. Nous rapportons le cas d’un nouveau-né à terme ayant un prolapsus génital sans anomalies de système nerveux central pris en charge au CHU Mohammed VI Marrakech. Nous rapportons à travers cette observation les particularités cliniques et thérapeutiques de cette pathologie rarissime.

## Patient et observation

**Figure 1:**
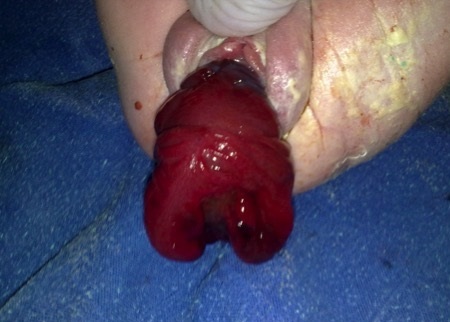
Prolapsus génital néonatal

Il s'agit d'un nouveau né de sexe féminin issu d'un mariage non consanguin chez une patiente primipare sans antécédents pathologiques particuliers, et menant une grossesse à terme non suivie. Le bébé pesait 2856g né par césarienne avec un Apgar 10/10. L'examen périnéal avait trouvé une masse rougeâtre prolabée par les grandes lèvres correspondant à un déroulement complet de la paroi vaginale et du col utérin (Figure 1). L'orifice urétral était normal, et le tonus sphinctérien anal était conservé. Le bilan malformatif clinique était négatif notamment pas de spina bifida ou autre anomalie rachidienne. L'examen neurologique retrouve un tonus axial et périphérique présents et les réflexes archaïques conservés. L'échographie abdomino-pelvienne n'a pas objectivé d'anomalie mise à part l'absence de visualisation de l'utérus. La TDM du rachis était normale. Le traitement a consisté en une réduction digitale du prolapsus avec une bonne évolution sans récidive avec un recul de 12 mois. L'évolution était bonne sans récidive sur une période de 12 mois.

## Discussion

Le prolapsus génital est une pathologie de la femme ménopausée, il reste exceptionnel chez le nouveau né [1]. Le premier cas a été décrit en Egypte dans le Papyrus Ebers 1500 avant le christ [2]. Le prolapsus néonatal est rare. Il est décrit à la naissance ou les premiers jours de la vie.il est associé à des anomalies congénitales du système nerveux dans 80% des cas, surtout les anomalies de fermeture du tube neurale [3]. Le diagnostic du prolapsus néonatal est clinique reposant sur la visualisation clinique d'une masse rouge ou rose faisant saillie à travers l'orifice vaginal [4], Correspondant à un déroulement circonférentiel de tout le mur vaginal. L'orifice cervical externe est généralement vu sur l'extrémité de la masse prolabée. L'orifice urétral est le plus souvent normal.il peut être associé à un prolapsus rectal [4, 5]. Le diagnostic différentiel de la masse interlabiale se pose devant les polypes vaginaux, le prolapsus urétral, les kystes para urétraux et le rhabdomyosarcome [5, 6]. L'échographie abdomino - pelvienne confirmera l'absence d'organes génitaux internes et recherchera d'autres anomalies associées [7]. La TDM et l'IRM rechercheront les anomalies médullaires notamment un spina bifida, ou une hydrocéphalie [8, 9]. Plusieurs modalités thérapeutiques ont été proposées, le traitement conservateur consiste en une réduction digitale avec l'inconvénient quelle soit répétée plusieurs fois. L'usage de la sonde de foley décrite par Ajabor et Okojie reste efficace en deuxième lieu après échec de la réduction pendant 2 semaines [10, 11]. Le traitement chirurgical reste exceptionnel, peut consister en une suture labiale, une cervicopexie sacrées ou une ventro suspension [12]. D'autres techniques telles que l'hystérectomie ou l'amputation cervicale de l'utérus sont historiques et ne devraient plus se voir. L'évolution est en générale favorable quand le prolapsus génital est isolé [12].

## Conclusion

Le prolapsus génital est une entité exceptionnelle chez le nouveau-né. Il est le plus souvent associé à des anomalies du système nerveux central. Le traitement est le plus souvent conservateur et le pronostic est en général favorable.
